# Reactivation of trauma in an inadequately structured person

**DOI:** 10.1192/j.eurpsy.2024.1386

**Published:** 2024-08-27

**Authors:** V. V. Jovanovska

**Affiliations:** Psychiatry, PHI General Hospital Kumanovo, Kumanovo, North Macedonia

## Abstract

**Introduction:**

The person is constantly exposed to various types of psychosocial stress, and what will be the course and outcome of the reaction, in addition to other factors, primarily depends on the structure of the person (cognitive, conative, affective and somatic characteristics).

**Objectives:**

Presentation of a case of an inadequately structured person (a 29-year-old girl) who experiences an emotional loss, thus reactivating a trauma experienced many years ago (content-like emotional loss). The activation of traumatic memory as a center for generating a complex of pathological symptoms is provoked due to the personal structural inability of a person to legally reorganize, reintegrate and absorb stress.

**Methods:**

For a complete psychological exploration of an organization, personality dynamics, symptoms, defenses, motives, goals, values, interpersonal relationships, etc. I have applied: MMPI-202, NEO PI-R, Millon’s test, PIE, ZS and Azinger aggression scale.

**Results:**

The result is an inadequately structured person of the avoidant type: introverted, vulnerable, disturbingly self-centered, constantly alert to prevent his impulses and affectional compunctions from leading to a repetition of pain and suffering experienced in the past, denying his feelings to maintain interpersonal distance, sensitive, helpless in an aggressive environment, low self-esteem and self-confidence, with weak capacities to overcome stress, etc. Anxiety and depressive symptoms are dominant. Manifest symptomatology is elaborated.

**Conclusions:**

In a person with an inadequate structure, there is an increased vulnerability, therefore applying an exploratory approach to people with a stressful condition in daily professional practice is a necessary need in order to more effectively, comprehensively treat the current and previously memorized stressful reactions with an emphasis on the highly personalized response to stress.

**Disclosure of Interest:**

None Declared

